# Effects of sodium benzoate on storage stability of previously improved beverage from tamarind (*Tamarindus indica* L.)

**DOI:** 10.1002/fsn3.78

**Published:** 2013-11-15

**Authors:** Abiodun A Adeola, Ogugua C Aworh

**Affiliations:** 1 Institute of Food Security, Environmental Resources and Agricultural Research, Federal University of AgricultureAbeokuta, Nigeria; 2 Department of Food Technology, University of IbadanIbadan, Nigeria

**Keywords:** Beverage, pasteurization, shelf life, tamarind

## Abstract

The effect of sodium benzoate on the quality attributes of improved tamarind beverage during storage was investigated. Tamarind beverages were produced according to a previously reported improved method, with or without chemical preservatives (100 mg/100 mL sodium benzoate). Tamarind beverage produced according to traditional processing method served as the control. The tamarind beverages were stored for 4 months at room (29 ± 2°C) and refrigerated (4–10°C) temperatures. Samples were analyzed, at regular intervals, for chemical, sensory, and microbiological qualities. Appearance of coliforms or overall acceptability score of 5.9 was used as deterioration index. The control beverages deteriorated by 2nd and 10th days at room and refrigerated temperatures, respectively. Improved tamarind beverage produced without the inclusion of sodium benzoate was stable for 3 and 5 weeks at room and refrigerated temperatures, respectively. Sodium benzoate extended the shelf life of the improved tamarind beverage to 6 and 13 weeks, respectively, at room and refrigerated temperatures.

## Introduction

Tamarind (*Tamarindus indica* L.), a tropical fruit found in Africa and Asia, is highly valued for its pulp (Gunasena and Hughes 2000). Tamarind fruit pulp has a sweet acidic taste due to a combination of high contents of tartaric acid and reducing sugars (De Caluwe et al. 2009). The pulp is used for seasoning, in prepared foods, to flavor confections, curries, and sauces, and as a major ingredient in juices and other beverages (De Caluwe et al. 2009).

Tamarind is commercially underexploited in Nigeria, where it is found growing wild on roadsides and uncultivated lands. In Nigeria, tamarind is known as “ajagbon” (Yoruba tribe) or “tsamiya” (Hausa tribe). While tamarind-based drinks are produced by industries in many countries where tamarinds grow, none exists in Nigeria. Tamarind fruits are only processed into beverage by indigenous people located in Northern Nigeria, despite the fact that tamarinds grow in some parts of southwest Nigeria. The production of tamarind beverage by the indigenous method is characterized by poor hygiene, labor-intensive manual operations, low aesthetic value, short shelf life and nonstandardization of ingredients, factors which have restricted the consumption of tamarind beverage to very few indigenous people in Northern Nigeria.

Adeola and Aworh (2010) had previously developed a process for the production of an improved beverage from Nigeria's tamarind. The process involved the removal of pulp from fruit, mixing with water, sieving, adding ginger, clove, and sugar, filling in bottles, and pasteurizing at 95°C for 8 min. However, they did not report on the shelf stability of the beverage, which is an important factor in the adoption of the improved method for commercial purpose.

This study therefore aimed at investigating the comparative effects of sodium benzoate and local spices (*Syzgium aromaticum* L. and *Zingiber officinale*) on the chemical, sensory, and microbial attributes during storage of tamarind beverage produced by the improved method.

## Material and Methods

### Materials

Mature tamarind (*T. indica* L.) fruits were obtained from Saki in Oyo State. Clove (*S. aromaticum* L.), ginger (*Z. officinale*), and granulated sugar were procured from a local market in Oyo town. All the chemicals used were produced by BDH (Lutterworth, U.K.).

### Production of tamarind beverages

**Figure 1:**
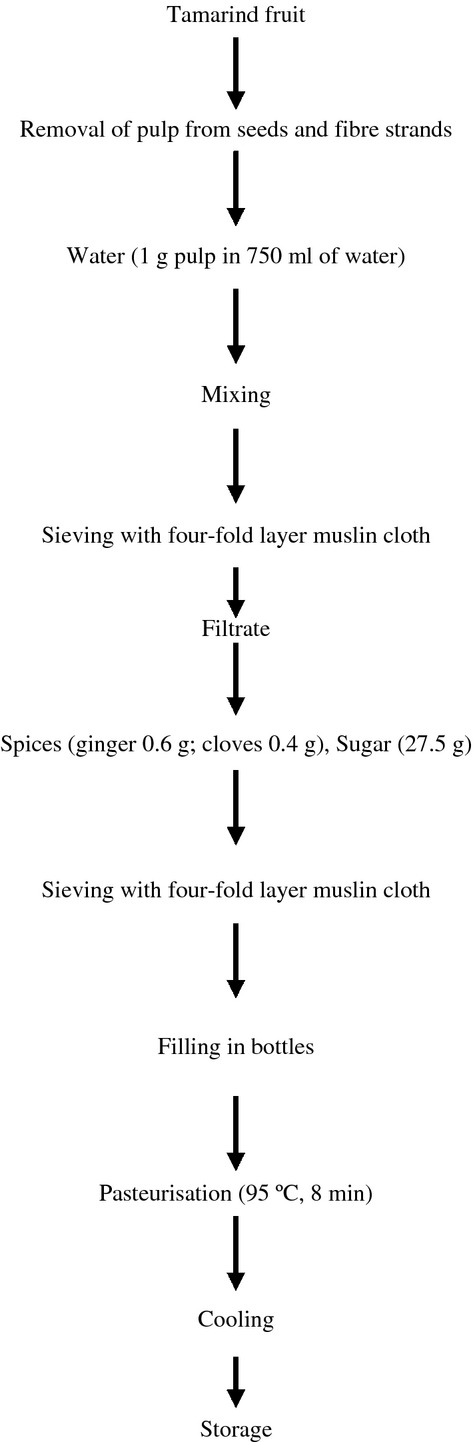
Improved processing of tamarind beverage. *Source*: Adeola and Aworh (2010).

Tamarind beverage was prepared according to the improved method of Adeola and Aworh (2010). Tamarind pulp was manually separated from shells, seeds, and other foreign materials. About 1 g of tamarind pulp was mixed with 750 mL of water. Spices (ginger 0.6 g, clove 0.4 g) and sugar (27.5 g) were added. The mixture was sieved with fourfold layer muslin cloth. The beverage was packaged in sterilized glass bottles, corked, and pasteurized at 95°C for 8 min (Fig. 1).

**Figure 2:**
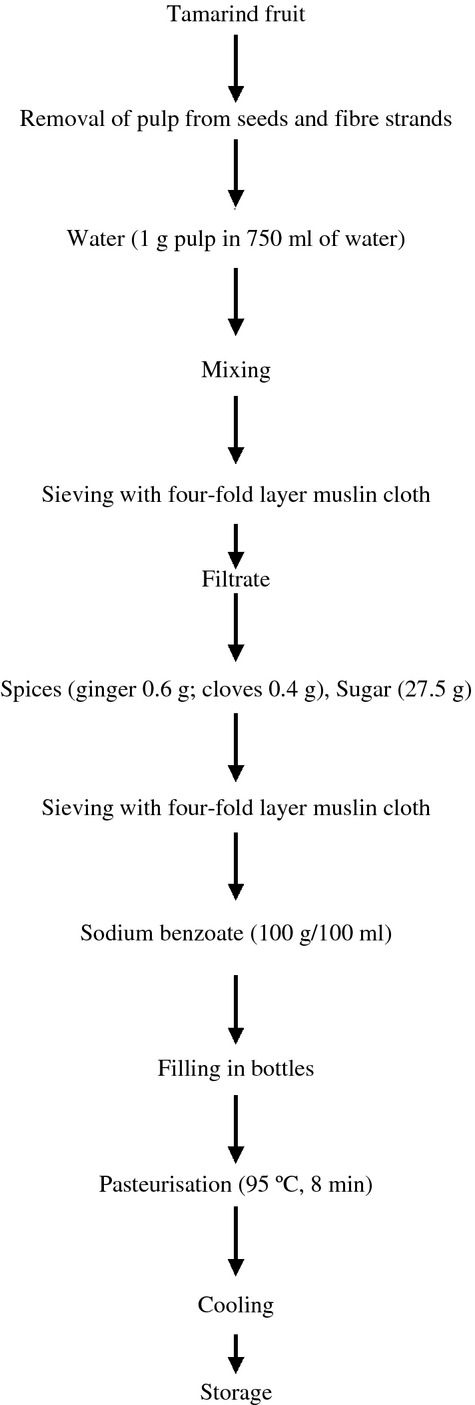
Improved processing of tamarind beverage containing sodium benzoate.

Furthermore, a pilot study was carried out in which the beverage was mixed with various concentrations of sodium benzoate (50–150 mg/100 mL). Paired preference test was used to determine the acceptable concentration of sodium benzoate, which was found to be 100 mg/100 mL. Consequently, improved tamarind beverage preserved with sodium benzoate was prepared according to Figure 2.

**Figure 3:**
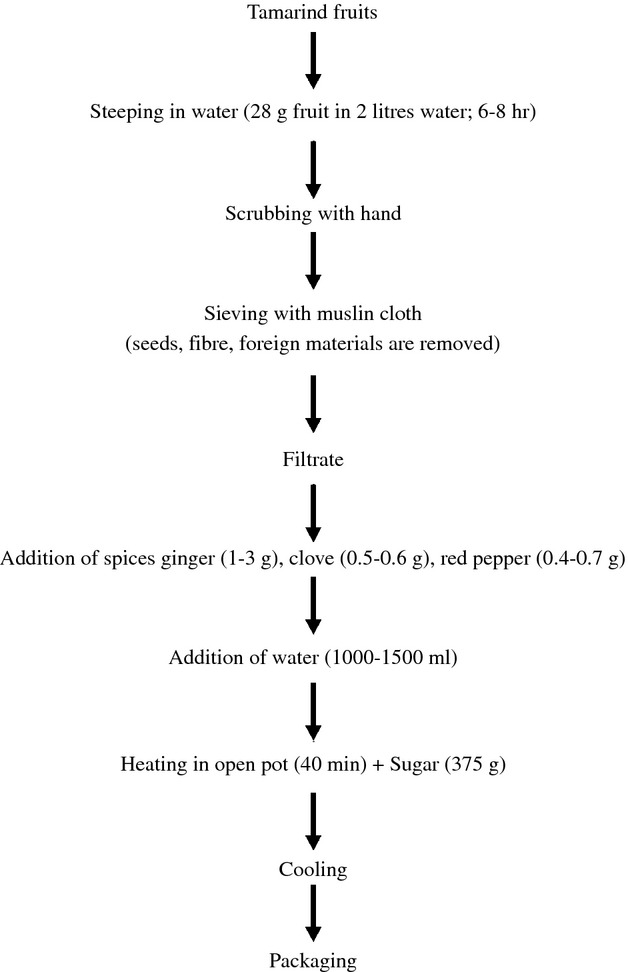
Traditional processing of tamarind beverage. *Source*: Adeola and Aworh (2010).

Tamarind beverage produced according to the traditional method (Fig. 3) was used as a reference.

### Storage

Tamarind beverages were stored at room (28 ± 2°C) and refrigerated (4–10°C) temperatures for 4 months. The beverage was assumed stable until the appearance of coliform or when the overall acceptance score was less than 5.9.

### Sensory evaluation

Tamarind beverages were evaluated by a panel of 20 assessors comprising of staff and students of Emmanuel Alayande College of Education, Oyo, using a 9-point hedonic scale, where 1 = *dislike extremely*, 5 = *neither like nor dislike*, and 9 = *like extremely*.

### Chemical analyses

The pH, total acidity, soluble solids, and total solids of tamarind beverages were determined according to A.O.A.C. (1995), ascorbic acid according to A.A.C.C (1983), color by measuring absorbance at 325 nm (Adeola and Aworh 2012), total solids according to Adeyemi and Umar (1994), browning index according to Johnson et al. (1995), and cloudiness according to Khurdiya and Verma (1996).

### Microbiological analyses

Samples were serially diluted in one-fourth strength Ringer's solution. Aliquots were further diluted to obtain 10^−1^ to 10^−4^ for microbial counts. About 1.0 mL was transferred aseptically to petri dishes, poured with plate count agar (PCA) for total viable counts, McConkey agar (MCC) for coliform counts, and acidified potato dextrose agar (PDA) for total molds and yeasts counts. PCA and MCC were incubated at 35°C for 48 h while PDA was incubated at 25°C for 3–5 days.

### Statistical analyses

Data obtained from chemical analyses were averaged and standard deviation calculated. Microbial counts were converted to log_10_ CFU/mL. One-way analysis of variance (ANOVA) was used for the analysis of the microbial logarithmic values and hedonic scores. Duncan's multiple range tests and least significant difference (LSD) were, respectively, used to separate means that were significantly different (*P* < 0.05) for microbial logarithmic values and hedonic scores.

## Result and Discussions

### Chemical properties

Table 1 shows the changes in the physical and chemical attributes during storage of tamarind beverages prepared by the traditional processing method. The beverage had a shelf life of 1 and 8 days at room and refrigerated temperatures, respectively. There were distinct changes in the chemical attributes of the beverage during storage at both temperatures, although the changes were less pronounced at refrigerated temperature. However, there was no significant change in the total acidity of the beverage up to 2 and 4 days of storage at room and refrigerated temperatures, respectively, while the pH changed significantly during the period. Paulson and Stevens (1974), Okoli and Ezenweke (1990) and Kamruzzaman et al. (2002) also observed that pH and acidity are not always inversely related.

**Table 1:** Physical and chemical attributes during storage of tamarind beverage produced by traditional processing method

Storage temperature	Period of storage (days)	Quality attributes									
		Color (*A*_325nm_)	Total solid (g/100 mL)	Total soluble solid (°Brix)	pH	Total acidity (%)	Cloudiness (*A*_660nm_)	Browning index (*A*_420nm_)	Total sugar (%)	Ascorbic acid (%)	Ash (%)
Room	0	0.91 ± 0.012	20.0 ± 0.15	19.5 ± 0.45	2.8 ±0.06	1.0 ±0.06	0.68 ± 0.02	1.42 ± 0.02	17.3 ± 0.53	9.4 ± 0.17	0.43 ± 0.058
Refrigerated	0	0.91 ± 0.012	20.0 ± 0.15	19.5 ± 0.45	2.8 ±0.06	1.0 ±0.06	0.68 ± 0.02	1.42 ±0.02	17.3 ± 0.53	9.4 ± 0.17	0.43 ± 0.058
	2	0.95 ± 0.006	15.4 ± 0.29	14.2 ± 0.3	2.6 ± 0.2	1.0 ± 0.08	0.65 ± 0.01	1.50 ± 0.02	13.4 ± 0.4	7.2 ± 0.35	0.37 ± 0.058
	4	0.96 ± 0.012	12.6 ± 0.21	11.4 ± 0.61	2.3 ± 0.06	1.0 ± 0.06	0.61 ± 0.006	1.51 ± 0.02	11.3 ± 0.57	6.1 ± 0.15	0.30 ± 0.003
	6	0.98 ± 0.006	10.1 ± 0.12	9.5 ± 0.4	2.0 ± 0.1	1.1 ± 0.1	0.54 ± 0.01	1.57 ± 0.02	8.6 ± 0.35	5.6 ± 0.26	0.30 ± 0.005
	8	0.98 ± 0.01	9.0 ± 0.17	8.5 ± 0.4	2.0 ± 0.06	1.1 ± 0.06	0.47 ± 0.006	1.62 ± 0.02	7.3 ± 0.56	5.1 ± 0.21	0.27 ± 0.057

Each result expresses mean ± SD.

The shelf life of the tamarind beverage produced by the improved processing method was 3 and 5 weeks at room and refrigerated temperatures, respectively (Table 2). There were significant differences in all the quality attributes except color and browning index, during storage. This observation is similar to that of Akubor and Abutu (2004) who also reported that not all the chemical constituents of melon–orange beverage changed during storage. The total acidity of the beverage did not change until weeks 2 and 4 of storage at room and refrigerated temperatures, respectively. The pH, on the other hand, changed throughout the storage period at room temperature while it only remained unchanged up to week 2 at refrigerated temperature.

**Table 2:** Physical and chemical attributes during storage of tamarind beverage produced by improved processing method

Storage temperature	Period of storage (days)	Quality attributes									
		Color (*A*_325nm_)	Total solid (g/100 mL)	Total soluble solid (°Brix)	pH	Total acidity (%)	Cloudiness (*A*_660nm_)	Browning index (*A*_420nm_)	Ascorbic acid (%)	Total sugar (%)	Ash (%)
Room	0	0.60 ± 0.012	10.8 ± 0.32	10.1 ± 0.12	3.4 ± 0.10	07 ± 0.06	0.13 ± 0.010	0.19 ± 0.010	10.4 ± 0.21	10.1 ± 0.23	0.23 ± 0.05
	1	0.60 ± 0.005	7.6 ± 0.20	7.0 ± 0.12	3.2 ± 0.20	0.7 ± 0.06	0.12 ± 0.006	0.19 ± 0.003	8.4 ± 0.20	6.7 ± 0.23	0.23 ± 0.08
	2	0.60 ± 0.025	5.8 ± 0.20	5.2 ± 0.31	3.0 ± 0.03	0.8 ± 0.05	0.11 ± 0.008	0.19 ± 0.010	7.1 ± 0.20	4.9 ± 0.40	0.10 ± 0.00
	3	0.62 ± 0.153	4.8 ± 0.06	4.1 ± 0.12	2.8 ± 0.10	1.0 ± 0.06	0.10 ± 0.004	0.20 ± 0.006	6.4 ± 0.30	3.9 ± 0.35	0.10 ± 0.00
Refrigerated	0	0.60 ± 0.012	10.8 ± 0.32	10.1 ± 0.12	3.4 ± 0.10	0.7 ± 0.06	0.13 ± 0.010	0.19 ± 0.010	10.4 ± 0.21	10.1 ± 0.23	0.23 ± 0.05
	1	0.60 ± 0.058	9.2 ± 0.15	9.1 ± 0.20	3.4 ± 0.10	0.7 ± 0.06	0.12 ± 0.006	0.19 ± 0.010	8.7 ± 0.21	8.5 ± 0.20	0.23 ± 0.05
	2	0.60 ± 0.006	7.8 ± 0.30	7.3 ± 0.17	3.4 ± 0.06	0.7 ± 0.03	0.11 ± 0.010	0.19 ± 0.010	7.8 ± 0.30	6.8 ± 0.06	0.10 ± 0.00
	3	0.60 ± 0.010	6.6 ± 0.21	6.4 ± 0.10	3.2 ± 0.12	0.7 ± 0.04	0.11 ± 0.010	0.19 ± 0.006	6.6 ± 0.31	5.5 ± 0.36	0.10 ± 0.00
	4	0.60 ± 0.006	6.1 ± 0.06	5.7 ± 0.40	3.0 ± 0.06	0.8 ± 0.06	0.10 ± 0.004	0.20 ± 0.007	6.1 ± 0.05	5.2 ± 0.25	0.10 ± 0.00
	5	0.60 ± 0.020	5.2 ± 0.15	4.8 ± 0.12	2.7 ± 0.10	1.0 ± 0.06	0.10 ± 0.004	0.20 ± 0.006	5.8 ± 0.30	4.3 ± 0.23	0.10 ± 0.00

Each result expresses mean ± SD.

The shelf life of the beverage preserved with 100 mg/100 mL sodium benzoate was 6 and 13 weeks at room and refrigerated temperatures, respectively (Table 3). The color, cloudiness, and browning index of the beverage were stable during storage at both room and refrigerated temperatures. Lee and Nagy (1988) advocated a lag period where changes in brightness were observed during storage of citrus juices. During this lag period, colorless compounds are probably formed which do not contribute to an increase in darkening. Thus, the improved processing method may have prolonged the lag period in tamarind beverages. The changes observed in the quality attributes of the tamarind beverage containing sodium benzoate were not as pronounced as the other beverages (Tables 1–3). There was no difference in the total acidity of the beverage until weeks 4 and 7 at room and refrigerated temperatures, respectively, while the pH only remained stable up to weeks 2 and 5 respectively at room and refrigerated temperatures. Thus, the improved beverage containing sodium benzoate had a higher buffering capacity than the other two tamarind beverages reported in Tables 1 and 3. Oliveira et al. (2013) also reported similar result during cold storage of acerola puree and suggested that processing and storage are viable alternatives to preserve the quality parameters of fruit juice acidity and pH.

**Table 3:** Physical and chemical attributes during storage of improved tamarind beverage containing 100 mg/100 mL sodium benzoate

Storage temperature	Period of storage (days)	Quality attributes									
		Color (*A*_325nm_)	Total solid (g/100 mL)	Total soluble solid (°Brix)	pH	Total acidity (%)	Cloudiness (*A*_660nm_)	Browning index (*A*_420nm_)	Ascorbic acid (%)	Total sugar (%)	Ash (%)
Room	0	0.54 ± 0.008	11.9 ± 0.20	11.6 ± 0.10	3.3 ± 0.10	0.7 ± 0.06	0.10 ± 0.007	0.17 ± 0.020	11.6 ± 0.17	11.5 ± 0.15	0.20 ± 0.008
	1	0.54 ± 0.030	10.8 ± 0.20	10.1 ± 0.30	3.3 ± 0.20	0.7 ± 0.10	0.10 ± 0.006	0.17 ± 0.010	11.0 ± 0.20	9.8 ± 0.36	0.20 ± 0.003
	2	0.54 ± 0.020	10.3 ± 0.30	9.9 ± 0.15	3.3 ± 0.20	0.7 ± 0.20	0.10 ± 0.005	0.17 ± 0.003	10.2 ± 0.40	9.6 ± 0.17	0.20 ± 0.006
	3	0.54 ± 0.040	9.2 ± 0.20	8.8 ± 0.25	3.2 ± 0.04	0.7 ± 0.06	0.10 ± 0.010	0.17 ± 0.006	9.4 ± 0.17	8.4 ± 0.20	0.20 ± 0.004
	4	0.56 ± 0.010	8.4 ± 0.12	8.0 ± 0.10	3.1 ± 0.30	1.0 ± 0.30	0.10 ± 0.006	0.17 ± 0.020	8.6 ± 0.45	7.4 ± 0.21	0.20 ± 0.006
	5	0.56 ± 0.012	7.1 ± 0.40	6.7 ± 0.25	3.1 ± 0.20	1.0 ± 0.20	0.10 ± 0.020	0.17 ± 0.010	7.8 ± 0.45	6.0 ± 0.10	0.20 ± 0.002
	6	0.56 ± 0.030	5.8 ± 0.12	5.2 ± 0.20	3.0 ± 0.04	1.0 ± 0.10	0.09 ± 0.010	0.18 ± 0.010	6.7 ± 0.23	4.9 ± 0.20	0.20 ± 0.004
Refrigerated	0	0.54 ± 0.008	11.9 ± 0.20	11.6 ± 0.10	3.3 ± 0.10	0.7 ± 0.06	0.10 ± 0.007	0.17 ± 0.020	11.6 ± 0.17	11.5 ± 0.15	0.20 ± 0.008
	1	0.54 ± 0.020	11.7 ± 030	11.6 ± 0.30	3.3 ± 0.20	0.7 ± 0.10	0.010 ± 0.006	0.17 ± 0.04	11.6 ± 0.23	11.2 ± 0.17	0.20 ± 0.006
	2	0.54 ± 0.020	11.5 ± 0.17	11.2 ± 0.23	3.3 ± 0.20	0.7 ± 0.20	0.10 ± 0.010	0.17 ± 0.010	11.4 ± 0.12	10.7 ± 0.17	0.20 ± 0.003
	3	0.54 ± 0.030	11.4 ± 0.40	11.1 ± 0.10	3.3 ± 0.20	0.7 ± 0.20	0.10 ± 0.004	0.17 ± 0.006	11.1 ± 0.30	107 ± 0.12	0.20 ± 0.042
	4	0.54 ± 0.010	11.2 ± 020	11.0 ± 0.06	3.3 ± 0.10	0.7 ± 0.06	0.10 ± 0.020	0.17 ± 0.010	10.9 ± 0.20	10.7 ± 0.06	0.20 ± 0.024
	5	0.54 ± 0.006	11.2 ± 0.06	10.9 ± 0.30	3.3 ± 0.30	0.7 ± 0.10	0.10 ± 0.010	0.17 ± 0.020	10.1 ± 0.12	10.1 ± 0.12	0.20 ± 0.008
	6	0.54 ± 0.006	11.0 ± 0.06	10.7 ± 0.12	3.2 ± 0.10	0.7 ± 0.10	0.10 ± 0.020	0.14 ± 0.005	9.8 ± 0.20	9.8 ± 0.15	0.20 ± 0.006
	7	0.54 ± 0.020	11.0 ± 0.40	10.8 ± 0.25	3.2 ± 0.20	0.7 ± 0.10	0.10 ± 0.046	0.17 ± 0.010	9.7 ± 0.30	9.5 ± 0.20	0.20 ± 0.004
	8	0.55 ± 0.030	10.5 ± 0.30	9.8 ± 0.06	3.2 ± 0.20	1.0 ± 0.06	0.10 ± 0.041	0.17 ± 0.005	9.3 ± 0.25	9.4 ± 0.17	0.20 ± 0.042
	9	0.55 ± 0.010	10.2 ± 0.20	9.1 ± 0.12	3.1 ± 0.20	1.0 ± 0.30	0.10 ± 0.041	0.17 ± 0.008	8.8 ± 0.30	9.2 ± 0.26	0.20 ± 0.005
	10	0.56 ± 0.020	9.8 ± 0.25	8.8 ± 0.35	3.1 ± 0.06	1.0 ± 0.08	0.09 ± 0.09	0.18 ± 0.030	8.5 ± 0.20	8.5 ± 0.30	0.20 ± 0.002
	11	0.57 ± 0.010	8.6 ± 0.12	8.1 ± 0.06	3.1 ± 0.20	1.0 ± 0.10	0.09 ± 0.020	0.18 ± 0.010	8.0 ± 0.25	7.7 ± 0.17	0.20 ± 0.004
	12	0.57 ± 0.020	7.8 ± 0.15	7.2 ± 0.20	3.0 ± 0.06	1.0 ± 0.20	0.09 ± 0.010	0.18 ± 0.007	7.1 ± 0.12	7.0 ± 0.30	0.20 ± 0.006
	13	0.58 ± 0.030	6.4 ± 0.20	5.9 ± 0.12	2.9 ± 0.20	1.0 ± 0.10	0.09 ± 0.04	0.19 ± 0.020	6.7 ± 0.31	5.8 ± 0.23	0.20 ± 0.008

Each result expresses mean ± SD.

Tables 1–3 revealed that refrigeration and chemical preservative (sodium benzoate) improved the keeping qualities of the tamarind beverages. Kabasakalis et al. (2000), Kamruzzaman et al. (2002), Onuorah et al. (2002), Akubor and Abutu (2004), and Ade-Omowaye et al. (2006) also observed similar results in some traditional and commercial beverages. Furthermore, the total acidity, color, and browning index of the beverages increased while the pH, cloudiness, ash, total solids, total soluble solids, ascorbic acid, and total sugar decreased during storage. Similar results were reported by Onuorah et al. (2002), Osundahunsi (2003), Supraditareporn and Pinthong (2007), Shende et al. (2009), and Oliveira et al. (2013). There were distinct differences in the quality attributes of the beverages, which may be as result of the differences in the processing methods. Akpapunam et al. (1993), Akinwale et al. (2001), and Balaswamy et al. (2011) reported that processing treatments affected the chemical constituents of fruit juices. Beverages produced by the improved processing method had lower sugar contents than the one produced by traditional processing method. This conforms to the recommendations of WHO Global Strategy on Diet, Physical activity and Health that levels of sugar, among some other nutrients, in manufactured food products are reduced (Roodenburg and Leenen 2007).

The high total solids recorded for the traditional beverage may be attributed to the increased sugar addition owing to its high sourness requiring more sweetener than the improved beverage which is less sour. The improved processing method resulted in an improvement in the color and ascorbic acid contents of beverages, probably due to the less severe effect of pasteurization on these quality attributes since carotenoids, *β*-carotene and lycopene in tamarind pulp could have decomposed to affect the color of beverage during heating in the traditional processing method. Gomez-Palomares et al. (2005) had earlier reported that pasteurization temperature of 90°C assured pectinesterase inactivation without detectable changes in flavor and color of tamarind nectar and puree. Furthermore, Sims et al. (1993) reported that blanching improved the color of carrot juice. The loss of ascorbic acid during storage may be due to catalytic degradation by ascorbate oxidase and peroxidase (Oliveira et al. 2013). The loss of cloud during storage of beverages produced by the improved processing method may be due to precipitation of pectin and other cell wall components. In all the beverages, a drop in total solids was accompanied by a decrease in soluble solid and sugar, implying that soluble solids make up a large percentage of total solids. The decrease in soluble solids can be attributed to the utilization of sucrose by microorganisms. Total solids thus decreased in part as a result of volatilization of soluble solids during metabolism. Browning was more pronounced in the beverage produced by the traditional processing method than those by the improved processing method, probably due to the higher total solids, total soluble solids, and sugar in the traditional beverage. Browning in the beverages could have been due to Maillard-type reactions resulting from the presence of reducing sugars, proteins, and amino acids. The observation that browning in tamarind beverages increased during storage just as pH decreased is contradictory to the claim of Dougherty and Nelson (1974) that Maillard-type reactions decreased in rate with decreased pH. The observed trend in browning in this study may be due to caramelization, which is enhanced by reduced pH. However, due to the fact that product was heated for only a relatively short time, browning may further be attributed to the decomposition of ascorbic acid to furfural and 5-hydroxymethyl furfural (Braverman 1963; Nagy and Smoot 1977; Akhavan and Wrolstad 1980; Smoot and Nagy 1980; Lee and Nagy 1988). The lower the pH, the more readily this reaction occurs (Dougherty and Nelson 1974). Beverage produced by the traditional processing method was more acidic than those from improved processing method. Tables 1–3 show that the effect of pasteurization time on the color, total solids, soluble solids, sugar, ascorbic acid, cloudiness, and browning index of beverages was less pronounced in the beverage preserved with sodium benzoate. Arya et al. (1979) and Okoli and Ezenweke (1990) also reported similar results.

### Microbial load

Table 4 shows the microbial load during storage of spiced tamarind beverage produced by traditional processing method. The total plate and total molds and yeasts counts of the beverage were 3.98 log CFU/mL and 4.58 log CFU/mL at room temperature, respectively, and 3.98–5.25 log CFU/mL and 4.58–5.67 log CFU/mL, respectively, at refrigerated temperature. No coliform bacteria were observed in the beverage until days 2 and 10 during storage at room and refrigerated temperatures, respectively. Onuorah et al. (2002) reported a microbial load of 1.5 × 10^3^ to 6.9 × 10^7^ CFU/mL and 1.5 × 10^3^ to 6.6 × 10^6^ CFU/mL for traditionally manufactured tamarind beverage stored at room and refrigerated temperatures, respectively. Osundahunsi (2003) reported an initial microbial load of 0.2 × 10^1^ CFU/mL for commercial samples of *soborodo*. This discrepancy may be due to differences in the initial microbial load and source of raw materials, processing methods, and levels of hygiene observed during production.

**Table 4:** Microbial load during storage of spiced tamarind beverage produced by traditional processing method

Storage temperature	Period of storage (days)	Microbial counts (log CFU/mL)		
		Total plate counts	Total molds and yeasts counts	Coliform counts
Room	0	3.98^a^	4.58^a^	NG
				NG
				NG
Refrigerated	0	3.98^a^	4.58^a^	NG
	2	4.58^ab^	5.70^b^	NG
	4	4.46^ab^	5.03^ab^	NG
	6	4.96^bc^	5.57^b^	NG
	8	5.25^c^	5.67^b^	NG

Means in the same column with same letters are not significant y different at the 5% level. NG, no growth.

The microbial load of tamarind beverage produced by the improved processing method is as shown in Table 5. The total plate count exceeded the total mold and yeast throughout the storage period of the beverage at both room and refrigerated temperatures. Coliform bacteria were not observed until weeks 4 and 6 at room and refrigerated temperatures, respectively, by which period the beverage was assumed unfit for human consumption. The microbial population of the beverage increased during storage at room and refrigerated temperatures. Table 6 shows the microbial population of the improved beverage containing 100 mg/100 mL sodium benzoate. The microbial load is lower than the other two beverages (Tables 4 and 5). Bacterial growth was delayed until weeks 3 and 5 at room and refrigerated temperatures, respectively.

**Table 5:** Microbial load during storage of tamarind beverage produced by improved processing method

Storage temperature	Period storage (weeks)	Microbial counts (log CFU/mL)		
		Total plate counts	Total molds and yeasts counts	Coliform counts
Room	0	2.15^a^	NG	NG
	1	2.25^bc^	1.45^c^	NG
	2	2.36^cd^	1.55^c^	NG
	3	2.42^d^	1.77^e^	NG
Refrigerated	0	2.15^a^	NG	NG
	1	2.16^a^	NG	NG
	2	2.13^a^	1.06^a^	NG
	3	2.21^ab^	1.33^b^	NG
	4	2.33^bc^	1.50^c^	NG
	5	2.40^d^	1.66^d^	NG

Means in the same column with same letters are not significantly different at the 5% level. NG, no growth.

**Table 6:** Microbial load during storage of improved tamarind beverage containing 100 mg/100 mL sodium benzoate

Storage temperature	Period of storage (weeks)	Microbial counts (log CFU/mL)	
		Total plate counts	Total molds and yeasts counts
Room	0	NG	NG
	1	NG	NG
	2	NG	NG
	3	1.51^e^	1.10^ab^
	4	1.33^bcde^	1.10^ab^
	5	1.42^cde^	1.10^ab^
	6	1.49^de^	1.10^ab^
Refrigerated	0	NG	NG
	1	NG	NG
	2	NG	NG
	3	NG	NG
	4	NG	NG
	5	1.06^a^	1.05^a^
	6	1.13^ab^	1.05^a^
	7	1.13^ab^	1.20^ab^
	8	1.24^abc^	1.08^a^
	9	1.28^bcd^	1.08^a^
	10	1.32^bcde^	1.10^ab^
	11	1.39^cde^	1.16^ab^
	12	1.46^de^	1.22^ab^
	13	1.50^e^	1.30^b^

Means in the same column with same letters are not significantly different at 5% level. NG, no growth.

Generally, tamarind beverage produced by the traditional processing method had higher microbial load than the ones produced by the improved processing method. This may be due to the fact that tamarind beverage, like many other traditional Nigerian beverages, is produced in homes and little attention is given to hygienic rules in the selection of processing materials and activities. The beverage is therefore highly susceptible to contamination by the microflora of the raw materials, utensils, and environment. The processing and storage conditions associated with traditional processing methods can encourage the presence of pathogenic or spoilage microorganisms in the beverage. Adeyemi and Umar (1994), Onuorah et al. (2002), and Ade-Omowaye et al. (2006) also reported high microbial counts in nonalcoholic traditional beverages. Refrigeration caused a further decline in the microbial load of the beverage. The low microbial counts of the pasteurized and refrigerated beverage samples may be due to the fact that pasteurization temperature can destroy the mesophilic microorganisms while low temperature can control the proliferation of those present. Okoli and Ezenweke (1990), Akpapunam et al. (1993), Adeyemi and Umar (1994), Oyawoye et al. (1997), Kamruzzaman et al. (2002), Onuorah et al. (2002), Osundahunsi (2003), and Osuntogun and Aboaba (2004) reported that pasteurization and refrigeration reduced the microbial population and extended the shelf life of traditional nonalcoholic beverages. The marked increase in the shelf life of the beverage when it was preserved with sodium benzoate agreed with the observation of Oyawoye et al. (1997). Chemical preservatives inhibit microorganisms by interfering with their cell membranes, enzyme activity or genetic mechanisms (Frazier and Westhoff 1978; Gasu et al. 2012). Some bacterial species were still observed as the acidic condition of the beverage increased during storage. Rhodes and Fletcher (1966) reported that some bacterial species were readily found growing in foods of low acid content where they produced organic acids.

### Sensory qualities

Tables 7–9 show the result of the sensory evaluation of tamarind beverages produced by the traditional and improved processing methods. Tamarind beverage produced by the traditional processing method was initially rated “like slightly” for color (6.1), taste (6.2), and overall acceptability, and “like much” (6.5) for aroma (Table7). During storage at room temperature, the sensory scores for all the attributes of the beverage produced by the traditional processing method decreased to “neither like nor dislike” on day 4. At refrigerated temperature, the samples were rated “neither like nor dislike” for all the quality attributes except aroma, which was rated dislike slightly at the end of storage life. Tamarind beverage produced by the improved processing method was initially scored “like very much” for color (7.7), aroma (7.8), and overall acceptability (7.7) and “like much” (7.4) for taste (Table 8). These scores reduced to “like slightly” for color (6.2), taste (6.4), and overall acceptability (6.2) and “like much” for aroma on week 3 at room temperature during storage. At refrigerated temperature, on the other hand, the beverage was rated “like slightly” for all the sensory attributes on week 5. Tamarind beverage preserved with sodium benzoate was initially rated “like very much” for all the sensory attributes. The rating thereafter decreased to “like slightly” for all the attributes at the end of storage at room and refrigerated temperatures (Table 9). Thus, the sensory scores of the tamarind beverages produced by the improved processing method were higher than the one produced by the traditional processing method. Sodium benzoate improved the sensory qualities of tamarind beverage. Akubor and Abutu (2004) also reported similar observation.

**Table 7:** Mean hedonic scores during storage of spiced tamarind beverage produced by traditional processing method

Storage temperature	Period of storage (days)	Mean hedonic scores			
		Color	Aroma	Taste	Overall acceptability
Room	0	6.1^a^	6.5^a^	6.2^a^	5.9^a^
Refrigerated	0	6.1^a^	6.5^a^	6.2^a^	5.9^a^
	2	6.1^a^	6.2^a^	6.0^ab^	5.8^a^
	4	5.8^ab^	5.9^b^	5.8^bc^	5.7^ab^
	6	5.5^b^	5.7^b^	5.5^ab^	5.3^ab^
	8	5.0^c^	5.1^d^	5.2^d^	5.2^ab^
	LSD	0.4	0.5	0.4	0.8

Means in the same column with same letters are not significantly different at the 5% level. LSD, least significant difference.

**Table 8:** Mean hedonic scores during storage of tamarind beverage produced by improved processing method

Storage temperature	Period of storage (weeks)	Mean hedonic scores			
		Color	Aroma	Taste	Overall acceptability
Room	0	7.7^a^	7.8^a^	7.4^a^	7.7^a^
	1	7.3^ab^	7.4^ab^	7.4^a^	7.1^abc^
	2	6.8^bcd^	7.1^abc^	7.0^bc^	6.6^cde^
	3	6.2^de^	6.9^bc^	6.4^cd^	6.2^de^
Refrigerated	0	7.7^a^	7.8^a^	7.4^a^	7.7^a^
	1	7.6^a^	7.6^ab^	7.4^a^	7.4^ab^
	2	7.3^ab^	7.3^abc^	7.2^ab^	6.9^bcd^
	3	6.9^bc^	7.2^abc^	6.9^abcd^	6.6^cde^
	4	6.5^cde^	7.0^bc^	6.7^bcd^	6.4^cde^
	5	6.0^e^	6.6^c^	6.3^d^	6.0^e^
	LSD	0.7	0.8	0.7	0.8

Means in the same column with same letters are not significantly different at the 5% level.

**Table 9:** Mean hedonic scores during storage of improved tamarind beverage containing 100 mg/100 mL sodium benzoate

Storage temperature	Period of storage (weeks)	Mean hedonic scores			
		Color	Taste	Aroma	Overall acceptability
Room	0	7.8^ab^	8.0^a^	8.0^a^	8.0^a^
	1	7.1^ab^	7.7^ab^	7.6^abcd^	7.6^abcd^
	2	6.9^bcd^	7.5^abc^	7.4^abcde^	7.3^abcde^
	3	6.7^bcd^	6.8^cde^	7.1^abcdef^	7.0^bcdefg^
	4	6.4^cd^	6.4^et^	6.7^cdet^	6.8^cdefg^
	5	6.3^cd^	6.1^ef^	6.5^ef^	6.5^efghi^
	6	6.1^d^	6.0^t^	6.3^t^	6.2^gh^
Refrigerated	0	7.8^ab^	8.0^a^	8.0^a^	8.0^a^
	1	7.8^ab^	8.0^a^	8.0^a^	8.0^a^
	2	7.5^ab^	8.0^a^	8.0^a^	8.0^a^
	3	7.5^ab^	7.8^ab^	7.8^ab^	8.0^a^
	4	7.5^ab^	7.7^ab^	7.8^ab^	7.8^ab^
	5	7.1^abc^	7.5^abc^	7.7^abc^	7.7^abc^
	6	7.0^abc^	7.2^bcd^	7.6^bcd^	7.7^abc^
	7	6.9^bcd^	7.1^bcd^	7.5^abcde^	7.5^abcd^
	8	6.8^bcd^	6.8^bcd^	7.3^abcdet^	7.2^abcdet^
	9	6.8^bcd^	6.6^det^	7.1^abcdet^	6.7^detghi^
	10	6.5 ^cd^	6.4^ef^	7.0^bcdet^	6.4^fghi^
	11	6.4 ^cd^	6.2^et^	6.8^bcdet^	6.2^ghd^
	12	6.3 ^cd^	6.0^f^	6.6^def^	6.1^hi^
	13	6.1^d^	5.9^t^	6.5^et^	6.0^i^
	LSD	0.9	0.8	1.1	0.9

Means in the same column with same letters are not significantly different at the 5% level.

## Conclusion

The inclusion of sodium benzoate at 100 mg/100 mL in the processing of tamarind beverage by the previously improved method enhanced the sensory qualities and shelf life of the beverage. The total acidity, color, and browning index of beverages increase while the pH, cloudiness, ash, total solids, soluble solids, ascorbic acid, and total sugar decrease during storage.

## Conflict of Interest

None declared.
